# Ni^2+^ and Cd^2+^ Biosorption Capacity and Redox-Mediated Toxicity Reduction in Bacterial Strains from Highly Contaminated Soils of Uzbekistan

**DOI:** 10.3390/microorganisms13071485

**Published:** 2025-06-26

**Authors:** Aziza Usmonkulova, Eligio Malusa, Gulchekhra Kadirova, Ilkhom Khalilov, Loredana Canfora, Liliya Abdulmyanova

**Affiliations:** 1 Institute of Microbiology of Academy Sciences of the Republic of Uzbekistan, st. A. Kadyri 7B, 100128 Tashkent, Uzbekistan; kadirovagul@mail.ru (G.K.); isko.khalil@mail.ru (I.K.); a_l_i_2020@mail.ru (L.A.); 2 Scientific Research Institute of the Plant Protection and Quarantine, st. Bobur 4., 111215 Tashkent, Uzbekistan; 3 Council for Agriculture Research and Economics, Research Center for Viticulture and Enology, via P. Micca 35, 14100 Asti, Italy; 4 Council for Agriculture Research and Economics, Research Center for Agriculture and Environment, via della Navicella 2-4, 00184 Rome, Italy; loredana.canfora@crea.gov.it

**Keywords:** nickel, cadmium, oxidation state, biosorption capacity, IR spectroscopy, resistant bacteria

## Abstract

In this study, Ni^2+^ and Cd^2+^ resistant *Pseudomonas aeruginosa* 18, *Enterobacter ludwigii* 11Uz, and *Enterobacter cloacae* Uz_5 strains were isolated from soils contaminated with heavy metals in the Samarkand and Kashkadarya regions (Uzbekistan), and tested to remove Ni^2+^ and Cd^2+^ ions from the environment via biosorption. The biosorption capacity of these strains was observed under in vitro conditions. The biosorption process was highly dependent on the growing conditions, with the highest biosorption rate observed after 300 min of incubation at pH 7.0, and 40 °C. The presence of functional groups such as S=O, NH_2_, and COOH in the biosorbing microorganisms was confirmed by IR spectroscopy. The adsorption capacity decreased when the initial metal concentration was increased and was enhanced with higher microbial biomass. *Enterobacter ludwigii* 11Uz strain was found to alter the toxic oxidation state of Ni^2+^ and Cd^2+^ cations, while *Pseudomonas aeruginosa* 18 and *Enterobacter cloacae* Uz_5 strains reduced the toxicity of Ni^2+^ cations only by changing their oxidation state. It was confirmed in our studies that the three selected bacterial strains actively participated in the detoxification of Cd^2+^ through the synthesis of cysteine amino acid.

## 1. Introduction

Heavy metal pollution is a significant concern due to its persistence and non-biodegradable nature. Soil pollution with heavy metals, especially in Uzbekistan, occurs mainly as a result of amounts of pesticides and contaminants being present in mineral fertilizers. Soil surveys carried out in various locations in Uzbekistan pointed out that the highest amount of copper (1580 mg/kg, i.e., 526 times higher than the permissible limit—PL) and molybdenum (639 mg/kg, i.e., about 64 times higher than the PL) were observed in the soil collected from an old agricultural field (Tashkent region) [1]. Increased concentrations of heavy metals such as zinc, lead, copper, and chromium have been measured in the vicinity of solid waste landfills in the Tashkent region [2]. Analysis of heavy metal soil pollution in the Jizzakh region showed a slight increase in the PL for copper, zinc, chromium, nickel, cobalt, and arsenic [3]. In the soils of the Kashkadarya region of Uzbekistan, the lead content and chromium content exceed the maximum PL by 1.4–1.5 times and 1.16 times, respectively [1]. The Ni content in irrigated soils of that region was on average 7.25 or 10.30 times higher than the PL in the 0–30 cm and 51–80 cm layers, respectively. The Cd content in the deep layer of soil (51–80 cm) was 1.5 times higher than the PL (0.75 mg/kg) [4]. Ni content in the soil upper layer (0–30 cm) of pastures is about 1.5 times higher than the PL, providing evidence that mineral fertilizers are among the main source of heavy metal pollution. Since Cd is one of the most dangerous metals for human health and Ni is found in high amounts in Uzbek soils, these two pollutants were considered for studying microbiological remediation in the present work.

Currently, remediation strategies are classified into two main categories, namely **passive** and **active**. Active methods—including soil washing, chemical extraction, and electrokinetic remediation—can be quickly applied and easier to control but often require significant financial investment, large amounts of reagents, and may generate toxic secondary wastes [5]. In contrast, passive methods, such as bioremediation utilizing microorganisms, plants (phytoremediation), or enzymatic processes to degrade or immobilize heavy metals, have raised interest due to their ecological compatibility, cost-effectiveness, and potential for in situ application [6]. Combining microorganisms and plants or various types of microorganisms is a potentially more efficient approach, though its success depends on the species of organisms involved in the process [7]. However, their effectiveness can be limited by factors such as metal bioavailability, soil properties, and microbial activity [8].

Among the latter methods, microbial biosorption offers numerous advantages, including activity within a wide range of pH values and temperatures, strong adsorption capacity, ease of metal separation from the microorganism, cost-effectiveness, and environmental safety [9]. Microorganisms can selectively adsorb both low and high concentrations of heavy metal ions [10], a process which is primarily governed by physical adsorption, ion exchange, complex formation, and bioaccumulation. Due to its negative charge, the cell wall is the main structural component of bacteria cells that interacts with metal ions such as Cd^2+^ and Ni^2+^ ions, leading to their immobilization [11]. Therefore, biosorbed metals can also be recovered and their usefulness recovered, a process that cannot occur for those separated by chemical or physical methods [12].

Biosorption efficiency is higher in living bacterial biomass compared to dead biomass and is typically dependent on nutrient availability, cell age, and other environmental factors [10]. However, live bacterial biomass is generally more prone to metal toxicity effects than dead bacterial biomass [12]. These effects are counterbalanced through various mechanisms, including the transformation, bioreduction, or biotransformation of heavy metals [13]. Furthermore, living cells can adapt to metal-contaminated environments by genetically altering their physiological, biochemical, and structural characteristics [14,15,16].

The reduction and oxidation of heavy metal ions by microorganisms can affect their solubility and toxicity, since these features are related to the metal oxidation state [17]. Chemolithotrophic bacteria play a major role in redox processes, enhancing the mobilization of metals [18]. Depending on the chemical characteristics of the environment, heavy metals can be oxidized, reduced, or precipitated into less toxic forms (e.g., sulfides or oxides) by enzymatic activity, which significantly contributes to the resistance of microorganisms to heavy metal toxicity [19,20,21]. A variety of soil bacteria that show resistance to various heavy metals, particularly species belonging to the genera *Pseudomonas*, *Bacillus*, *Rhizobium, Shewanella*, *and Desulfovibrio*, have been exploited for the bioremediation of contaminated soils [6]. They contribute to the detoxification of metals through mechanisms such as the biosorption, bioaccumulation, enzymatic transformation, precipitation, and reduction of metal ions to less toxic or immobilized forms [22]. To remain viable and active in a contaminated environment, these bacteria must adapt to the chemical and physical stresses caused by heavy metal poisoning. Viability is often supported by mechanisms such as efflux pumps, metal-binding proteins (e.g., metallothionins), and the formation of biofilms that protect against harsh conditions [23]. Once bacteria have accumulated heavy metals in their biomass, the fate of this biomass becomes a major concern for remediation efficiency and environmental safety [18,19,20].

Soils studied for microbial heavy metal remediation are characterized by a moderate pH (typically 6.0–8.0), porosity, sufficient moisture, and organic matter. Effective bioremediation depends not only on the bacterial species and their metabolic capacity but also on soil conditions that support microbial viability and optimize mass transfer processes [12,21].

In this study, experiments were carried out to identify the mechanism involved in the detoxification of Cd^2+^ and Ni^2+^ ions by three strains of Ni- and Cd-resistant bacteria that were isolated from polluted soils, with the aim to enhance the understanding and efficiency of bacterial bioremediation/biosorption and detoxification for use in practical applications. For this reason, the biosorption capacity was evaluated under varying environmental conditions (pH, temperature, incubation time, and biomass concentration). The findings could thus contribute to the optimization of biosorption parameters, essential for the development of sustainable and efficient bioremediation technologies (i.e., production and formulation of the strains and their application in polluted fields).

## 2. Materials and Methods

### 2.1. Bacteria Strains

Cd- and Ni-resistant strains were isolated from Cd- and Ni-contaminated soil located in the Kashkadarya region (38°20′36.1″ N 66°26′45.3″ E) and Samarkand Region (39°41′11.0″N 66°48′19.8″E. The isolated strains were identified by 16S rDNA sequence analysis and registered in the NCBI GenBank database as *Pseudomonas aeruginosa* 18, *Enterobacter ludwigii* 11Uz, and *Enterobacter cloacae* Uz_5. Currently, these strains are stored in the “Industrially Important Microorganisms Collection” of the Institute of Microbiology, Tashkent.

### 2.2. Preparation of Selected Bacteria Biomass

The strains were cultivated in a liquid peptone medium at 28 °C for 36 h on an orbital shaker (IKA KS 4000i control, Thermo Fisher Scientific Inc., Waltham, MA, USA) at 120 rpm; at the end of the exponential growth phase, bacterial cells were collected by centrifugation at 6000/8000 rpm for 5/15 min (Eppendorf 5810 R, Hamburg, Germany). The biomass was washed three times with a sterile phosphate-buffered solution (pH 7.2), followed by distilled water to remove the residual growth medium. The biomass was then used for the experiments.

### 2.3. Determination of Bacterial Biosorption Capacity Under Variable Environmental Conditions

Biosorption experiments were performed by incubating the bacterial cells with the cadmium chloride (CdCl_2_) or nickel sulfate (NiSO_4_ × 7H_2_O), at concentrations of 24.6 mg/L and 200 mg/L, respectively. These concentrations were selected to reflect both environmental relevance (50–80 cm layer of soils have of 0.75 mg/kg Cd, 41.2 mg/kg Ni) and relative toxicity: cadmium is significantly more toxic than nickel and is strictly regulated at lower permissible limits in environmental and health guidelines. The biosorption capacity was determined under varying environmental conditions, including various pH, temperature, incubation time, and cell biomass concentration. The effect of the initial pH on bacterial biosorption was evaluated in the range of 3.0 to 8.0. The pH of the biosorption medium was adjusted using 0.1 M HCl or NaOH solutions before the addition of bacterial cells. This range was chosen based on the known sensitivity of biosorption efficiency to pH, which influences both metal ion speciation and the ionization state of functional groups on the bacterial cell surface (e.g., carboxyl, hydroxyl, and phosphate groups). At lower pH values, competition from excess H^+^ ions can inhibit metal binding, while at higher pH values, metals may precipitate as hydroxides, distorting biosorption measurements. The temperature range for the biosorption experiments was set from 15 °C to 40 °C. The experiments were carried out in a temperature-controlled shaker incubator to ensure uniform temperature conditions. This range encompasses typical environmental conditions found in Uzbekistan regions characterized by polluted soils and allows for the evaluation of thermal effects on metal uptake kinetics and biomass integrity. The effect of incubation time on biosorption was tested at different time points: 6, 12, 24, 48, and 72 h. At each time interval, samples were withdrawn, and the residual metal concentration in the solution was measured.

The effect of cell biomass concentration on biosorption capacity was tested by varying the bacterial cell concentration between 0.5 g/L and 3.0 g/L. For each concentration, the biosorption experiment was carried out at the optimal temperature, pH, and incubation time as determined in the previous experiments.

For all biosorption experiments, various amounts of bacterial biomass were added to 25 mL of growth medium to which a known concentration of the specific metal ion was added. The mixture was incubated under constant shaking conditions (150 rpm) at the specified temperature. After the desired incubation time, the suspension was sampled and filtered through a 0.22 µm membrane, and the concentration of Ni^2+^/Cd^2+^ in the filtrate was measured using an optical emission spectrometer (Perkin Elmer Avio 200, Waltham, MA, USA). To determine metal loss related to processes other than biosorption, a control sample containing only the growth medium and the heavy metals solutions without bacterial biomass were used. The results were expressed as the bacterial biosorption percentage using the following equation:

Biosorption Percentage (%) = (Ci − Cf)/Ci × 100(1)

where Ci and Cf represent the initial and final concentrations of the heavy metals, respectively [12,20].

### 2.4. Study of Redox Process Reducing the Toxicity of Heavy Metal Cations

The ability of the selected bacteria strains to convert Cd^2+^ cations into the non-toxic CdS form was determined in Nutrient-Limited Medium (P2). Initially, the bacteria were cultivated in the P2 medium without cysteine and Cd salt at 37 °C for 24 h. Then, 1 mM of CdCl_2_ salt was added, and the mixture was incubated for 6 h. After 6 h of incubation, if a yellow-green precipitate was formed in the medium, it was concluded that the Cd^2+^ cations were converted to non-toxic CdS through a reaction of bacteria-produced amino acid cysteine, producing the colored sulfide group (CdS) [24].

### 2.5. Observation of Oxidation–Reduction of Ni^2+^ and Cd^2+^ by Bacteria

The change in the oxidation state of Ni^2+^ and Cd^2+^ as a result of the redox activity of the bacteria was determined colorimetrically [25]. The selected bacteria were cultured for 48 h in a modified nutrient peptone medium containing appropriate amounts of Ni^2+^ and Cd^2+^ based on the MIC (minimum inhibitory concentration). In our previous studies, the MIC values of *Pseudomonas aeruginosa* 18, *Enterobacter ludwigii* 11Uz, and *Enterobacter cloacae* Uz_5 strains against Ni^2+^ and Cd^2+^ cations were determined to be 560, 840, and 840 mg/L and 549, 183, and 183 mg/L, respectively. The MIC values of bacteria against metals were determined by the Agarwal method [26]. The supernatant was separated from the biomass by centrifugation. NaOH, Na_2_CO_3_, and Na_2_HPO_4_ were added to the separated supernatant in proportions corresponding to the amounts of Ni^2+^ and Cd^2+^ in the nutrient medium. These substances served as qualitative analysis indicators for Ni^2+^ and Cd^2+^ cations, as their interaction results in the formation of precipitates of various colors. The indicators form a green precipitate upon interaction with Ni^2+^ and a white precipitate upon exposure to Cd^2+^ cations [25]. Pure nutrient medium both with and without the addition of Ni^2+^ and Cd^2+^ (without bacterial biomass) was used as the control. The absence of precipitate formation was assessed to determine whether the oxidation state of the two cations had changed as a result of bacterial activity, and the toxic form of the metal was lost [27].

## 3. Results

### 3.1. Effect of Growing and Environmental Conditions on the Biosorption Capacity of the Bacterial Strains

**Figure 1:**
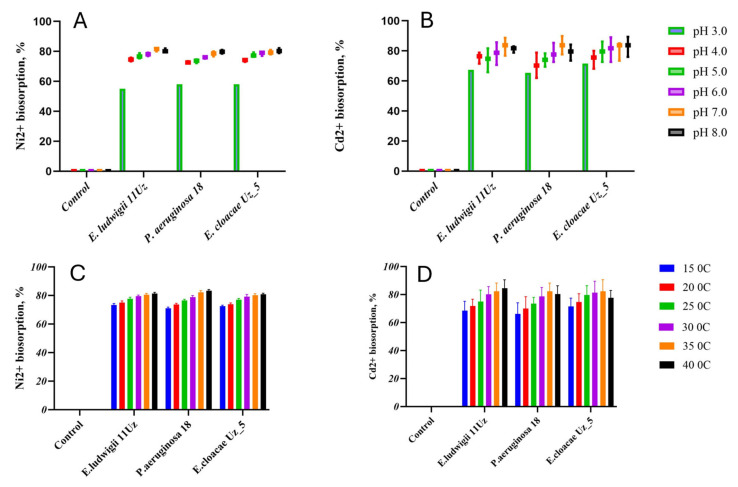
Ni^2+^ and Cd^2+^ biosorption capacity of the three selected bacteria strains as affected by various pH (**A**,**B**) and temperature (**C**,**D**) levels. Means ± SD.

The biosorption capacities of all strains for Ni^2+^ and Cd^2+^ were strongly affected by the pH of the solution, generally increasing with the transition from acid to neutral conditions, without further significant changes upon reaching alkaline pH (Figure 1A,B). The biosorption capacity of all strains ranged from 58% to 83% and 63.8% to 82.7% in cases of Ni^2+^ and Cd^2+^, respectively, depending on the pH. The removal of the heavy metal cations increased by about 10% and 20% for Ni^2+^ and Cd^2+^, respectively (Figure 1A,B).

No significant differences in the biosorption of Ni^2+^ and Cd^2+^ were observed between 15 °C and 20 °C, but the biosorption capacities of all strains increased with higher temperatures. This pattern was particularly noted with *Pseudomonas aeruginosa* 18, in which the biosorption capacity increased from 142 mg and 16.6 mg at 15 °C to 167 mg and 20.6 mg at 40 °C for Ni^2+^ and Cd^2+^, respectively. The 25 °C increase in the temperature thus induced thus an enhancement in the biosorption capacity of about 12% or 15% for Ni^2+^ and Cd^2+^, respectively (Figure 1C,D).

**Figure 2:**
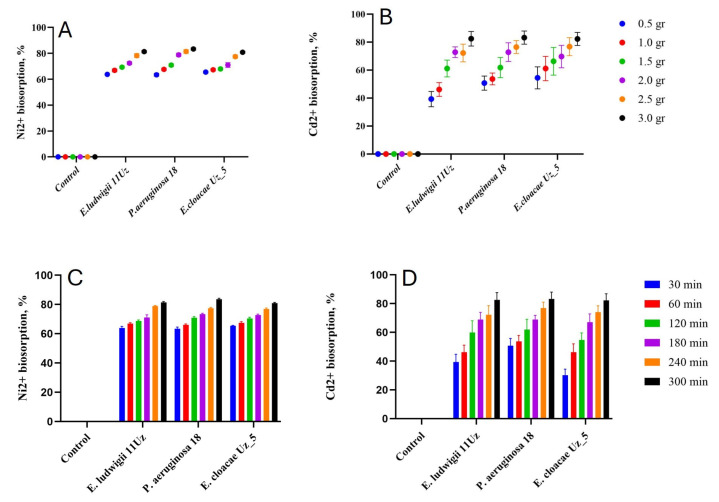
Ni^2+^ and Cd^2+^ biosorption capacity of the three selected bacteria strains as affected by biomass amounts (**A**,**B**) and incubation period (**C**,**D**). Means ± SD.

The efficiency of Ni^2+^ and Cd^2+^ removal was positively correlated to the amount of bacterial biomass (Figure 2). However, the relation was different for the two cations and was partly affected by the strain. *P. aeruginosa* 18 was able to increase the Ni^2+^ biosorption by about 10% when the biomass was increased six-fold, while this increase was more than 30% in case of Cd^2+^. On average, the three strains biosorption capacity was about 72% or 64% in case of Ni^2+^ and Cd^2+^, respectively.

The amount of biosorption gradually increased with the duration of the experiment, increasing after approximately 1 and 4 h for Ni^2+^ and Cd^2+^, respectively (Figure 2). All three strains responded similarly in terms of biosorption capacity in relation to the incubation time. However, Cd^2+^ biosorption was more efficient with much longer incubation time, allowing for reductions in the cation concentration of up to 85% in the solution.

### 3.2. Identification of Bacterial Functional Groups Responsible for Heavy Metal Binding

The bioadsorption of heavy metals on the cell surface can be carried out by simple physical methods without disrupting the structural integrity of the cells. FTIR spectroscopy helped in identifying the various functional groups present in bacterial cells in response to heavy metal stress.

**Figure 3:**
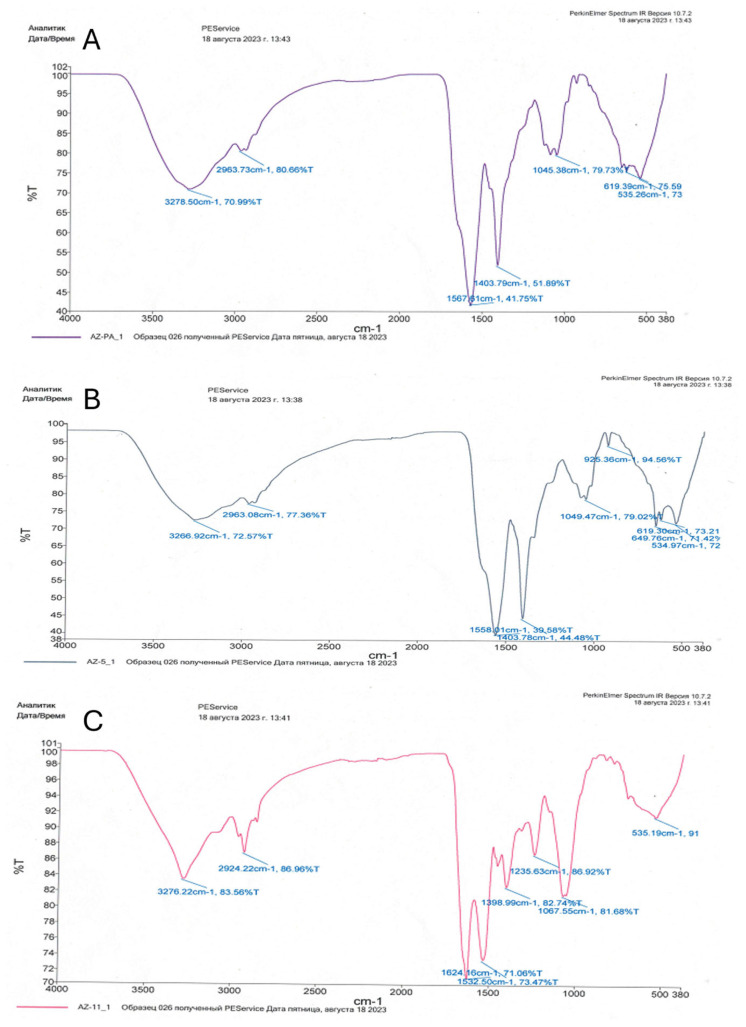
IR spectroscopy of bacterial biomass: (**A**)—*Pseudomonas aeruginosa* 18; (**B**)—*Enterobacter cloacae* Uz_5; (**C**)—*Enterobacter ludwigii* 11 Uz.

The functional groups detected with FTIR analysis were not always present in all three bacteria strains (Table 1, Figure 3). Alcoholic (C-OH), ketonic (C=O), amid (N-H), amino (NH_2_), and sulfoxide (S=O) groups were common to all. Interestingly, a diverse number of different amino groups was detected for the three strains. Acid (COOH) and aromatic (C-N) groups were detected only in *P. aeruginosa* and *E. cloacae* strains, while C-N and P-O groups were identified only in *E. ludwigii,* as indicated by the presence of specific peaks (Figure 3).

The C-S, CH_2_/CH_3_, -CH, and P=S groups, do not participate in the binding of heavy metal cations in bacterial biomass, and the presence of any these groups was not detected.

### 3.3. Monitoring the Redox Process That Reduces the Toxicity of Heavy Metal Cations

The addition of indicators led to the formation of green and white precipitates when no bacterial cells were present in the medium (control), indicating that the Ni^2+^ and Cd^2+^ ions in the nutrient medium were in the +2 oxidation state (Table 2).

*P. aeruginosa* 18 and *E. cloacae* Uz_5 strains were only able to alter the oxidation state of Ni^2+^ cations, changing them from the +2 oxidation state, while they did not affect the Cd^2+^ cations (Table 2). On the other hand, *E. ludwigii* 11Uz was able to detoxify the substrate by modifying the +2 oxidation state of both Ni^2+^ and Cd^2+^ cations.

After 6 h of incubation, a yellow-green precipitate was visually observed in the cultures of all three bacterial strains exposed to Cd^2+^. This observation suggested a possible involvement of cysteine synthesis in the detoxification process.

## 4. Discussion

The study showed that pH significantly influenced the biosorption capacity of all tested bacterial strains for both Ni^2+^ and Cd^2+^. The uptake of both metals increased as the pH shifted from acidic to neutral conditions. For both Ni^2+^ and Cd^2+^ biosorption increased by about 25%. Cd^2+^ biosorption exhibited a slightly higher sensitivity to pH change compared to Ni^2+^, increasing by 20% when changing from acidic to neutral/alkaline pH, compared to 10% for Ni^2+^. This suggests that Cd^2+^ biosorption may be more affected by changes in ion exchange capacity and surface charge distribution. This trend aligns with findings from previous studies, where pH has been proved to highly affect microorganisms’ biosorption efficiency of Cd^2+^ and Ni^2+^ [28,29]. This phenomenon can be explained by the competition between protons and Ni^2+^ and Cd^2+^ ions for the negatively charged functional group binding sites on the cell surface: as the pH increases, the number of protons decreases, and the number of binding sites for Ni^2+^ and Cd^2+^ increases [30,31]. Furthermore, as the pH increases, the deprotonation of binding sites, reduced electronic repulsion, and enhanced attraction of Ni^2+^ and Cd^2+^ cations may cause bacterial surface functional groups to become negatively charged [32,33]. Tests were not conducted at pH values above 8.0 due to the potential formation of metal hydroxide or oxide complexes as a result of hydrolysis under alkaline conditions.

Temperature is another crucial factor that influenced biosorption. No significant differences were found between 15 °C and 20 °C, but the capacities increased at higher temperatures, particularly in *P. aeruginosa* 18. This corresponds to an average increase in efficiency of ~12% and ~15% for Ni^2+^ and Cd^2+^, respectively. Importantly, the temperature-dependent trends were observed across all strains, highlighting a conserved biological response, although the strain-specific variation in the absolute uptake values suggests differences in thermotolerance or surface binding chemistry. Temperature-dependent biosorption enhancement has been reported in other microbial systems [34]. High temperatures can increase the kinetic energy of the solution and the surface activity, the diffusion rates, and, possibly, the permeability of cell walls, facilitating ion exchange [35]. However, excessive temperatures may denature cell wall proteins, limiting biosorption. The observed enhancement suggests that the process includes both passive surface binding and energy-dependent mechanisms and it has been proposed that the biosorption process is endothermic in nature [36].

The duration of exposure also affected metal removal efficiency, with equilibrium being reached faster for Ni^2+^ (1 h) than for Cd^2+^ (4 h). Cd^2+^ biosorption continued to improve with extended incubation, allowing up to 85% removal, which was ~50% higher than what was observed at 30 min. This could reflect a multi-phase binding process, including an initial rapid surface binding followed by a slower intracellular sequestration or complexation phase. Furthermore, the slower saturation for Cd^2+^ may reflect its larger ionic radius or lower diffusion coefficient, which has also been documented in other bacterial systems [9,36].

The biosorption capacity of *E. cloacae* Uz_5, *E. ludwigii* 11Uz, and *P. aeruginosa* 18 strains remained steady after 5 h of incubation, indicating that approximately 3 g/L of bacterial cells became saturated with Ni and Cd after 5 h, which can thus be considered the optimal immobilization time to assess such capacity. Therefore, the effect of bacterial biomass on Ni and Cd immobilization efficiency was measured at the optimal immobilization time until 6 h. The biosorption processes requiring a short time would be highly advantageous for practical applications. Slight fluctuations in the biosorption rate may result in the desorption or release of metal ions from the bacterial cells

The biosorption of both Ni^2+^ and Cd^2+^ was positively correlated with the amount of bacterial biomass, highlighting the importance of surface area and the density of available binding sites in determining biosorption capacity [37]. However, this relationship was not uniform across metals or bacterial strains. *P. aeruginosa* 18 demonstrated a notable increase in Cd^2+^ uptake with the increase in biomass—over 30%—while the same increase in biomass led to only a 10% improvement in Ni^2+^ uptake. This indicates that Cd^2+^ removal may benefit more from the availability of additional sorption sites, or that Cd^2+^ has a higher affinity for certain functional groups that become more abundant with increased biomass.

Increasing the bacterial cell biomass in a 150 mL Cd and Ni solution did not significantly improve Cd and Ni immobilization efficiency, indicating that a high quantity of microbial cells in a limited heavy metal solution does not enhance the immobilization of metal ions. Therefore, finding the optimal balance between the amount of biosorbent and the volume of the heavy metal solution is important. In this study, it was determined that 3 g/L of bacterial cells was the most optimal amount for a 150 mL Ni solution with a concentration of 200 mg/L.

Overall, the average biosorption capacities across all strains were higher for Ni^2+^ (72%) than for Cd^2+^ (64%). The variation between strains in terms of biosorption efficiency points to differences in cell surface chemistry and the presence of specific functional groups, as corroborated by infrared (IR) spectroscopy. IR spectroscopy has a high sensitivity for detecting functional groups and changes in bacterial components such as lipids and proteins [38].

Bacterial cells consist of lipids, proteins, and carbohydrates, which contain chemically active functional groups (such as PO_4_^3−^, OH^−^, S^2−^, CO_2_^−^, and SO_4_^2−^) known to contribute to metal ion chelation. These functional groups participate in metal-microbe interactions and are involved in the metal adsorption mechanism on bacterial surfaces [23,39]. For example, the presence of these functional groups was noted in *Bacillus* sp. MC3B-22, *Microbacterium* sp. MC3B-10, and *Bacillus* sp. strains, which were able to remove 75% of Cd^2+^ from the environment [40]. These functional groups including hydroxyl, carboxyl, phosphate, and amino can form electrostatic, ion-dipole, and coordination interactions with metal ions, leading to their immobilization on bacterial surfaces [41] and thus ensuring biosorption capacity [42].

The alteration of the oxidation–reduction state of heavy metal ions through reduction or oxidation reactions can effectively reduce their toxicity [43]. This protective mechanism can be managed by detoxifying enzymes regulated by the specific resistance genes of microorganisms. For example, bacteria like *Bacillus* sp. exhibit resistance to mercury ions through the action of mercury reductase [44].

Beyond passive biosorption, certain bacterial strains showed evidence of metabolic detoxification pathways—an ability to chemically alter or reduce the toxicity of heavy metals through redox transformation or bioprecipitation. The colorimetric redox assay indicated that *P. aeruginosa* 18 and *E. cloacae* Uz_5 could alter the oxidation state of Ni^2+^, but not Cd^2+^. In contrast, *E. ludwigii* 11Uz was capable of reducing both Ni^2+^ and Cd^2+^, indicating a broader detoxification capacity.

A promising pathway for Cd^2+^ detoxification involves the synthesis of non-toxic CdS, a sulfur-containing amino acid. The visual formation of yellow-green precipitates after 6 h of incubation with Cd^2+^ suggested the biosynthesis of cadmium sulfide (CdS), a relatively insoluble and less toxic compound [24]. This biotransformation is likely mediated by cysteine’s sulfhydryl (-SH) groups, which can bind with Cd^2+^ to form CdS nanoparticles [24,45]. Heavy metals form insoluble complexes with thiol groups, which play an important role in coordinating the antioxidant defense systems of living organisms. They are strong antioxidants that act as electron acceptors, reducing unstable free radicals through oxidation [46]. Important enzymes in microbial metabolism often contain sulfhydryl (SH) groups, and heavy metals such as Cd^2+^, Ag^2+^, and Hg^2+^ can bind to these groups, which inhibits the activity of metals [47]. On metal-resistant bacterial surfaces, anion groups such as S^2−^ and PO_4_^3−^ are present, which easily bind with Cd^2+^, thereby reducing the phytoavailability of metal ions [48]. Although not quantitatively confirmed in this study, this mechanism is consistent with the previous literature and warrants further validation to quantify cysteine production and CdS formation through spectrophotometric and molecular assay.

The findings presented in this work have clear implications for practical applications. The rapid biosorption kinetics (within 1–4 h), enhanced metal uptake under neutral to slightly alkaline conditions, and effective detoxification mechanisms, make these bacterial strains strong candidates for bio-based remediation technologies. For instance, the use of *E. ludwigii* in Cd^2+^-rich environments could be particularly beneficial due to its dual biosorption and redox conversion capabilities. Moreover, optimizing biomass dosage could enhance biosorption performance while minimizing the process costs in industrial-scale bioreactors, constructed wetlands, or in situ soil treatment.

## 5. Conclusions

This study highlights the significant potential of selected bacterial strains in the biosorption and detoxification of heavy metal ions, particularly Ni^2+^ and Cd^2+^. The experimental results demonstrated that the biosorption capacity of *Pseudomonas aeruginosa* 18, *Enterobacter ludwigii* 11Uz, and *Enterobacter cloacae* Uz_5 was influenced by various environmental factors such as pH, temperature, and biosorbent dosage. Among these strains, *Enterobacter ludwigii* 11Uz exhibited the most effective capacity to reduce the toxic 2+ oxidation state of Ni^2+^ and Cd^2+^ ions, transforming them into less harmful forms.

Additionally, cysteine played a crucial role in the detoxification process by facilitating the transformation of Cd^2+^ into a less toxic CdS precipitate. This suggests that the selected bacterial strains possess intrinsic mechanisms to not only biosorb but also detoxify heavy metals, making them promising candidates for bioremediation applications. The involvement of functional groups such as hydroxyl, amine, carboxyl, phosphate, and sulfur in the biosorption process further emphasizes the biochemical versatility of these bacteria.

These findings suggest that these bacterial strains isolated from soils in Uzbekistan can be effectively utilized for the remediation of environments contaminated with Ni^2+^ and Cd^2+^, offering a sustainable alternative to traditional methods of heavy metal removal. The formulation and application methods most suitable for exploiting these strains are currently under development. These local bacterial strains could be used as unexpensive, environmentally friendly, and efficient biosorbents for cleaning Ni^2+^ and Cd^2+^ contaminated environments without polluting the surroundings.

Based on these findings, future research should explore the molecular and genetic basis of heavy metal biosorption and redox transformation in these strains. Expanding the study to include mixed-metal systems and more complex real-world effluents will help assess the robustness and selectivity of these bacterial strains under environmentally relevant conditions. In this context, the development of a consortium with these strains with proper formulation may allow for the enhancement or combination of desirable traits across strains, which can be tailored for specific bioremediation applications in heavy metal-contaminated environments.

## Figures and Tables

**Table 1:** FTIR values determining the functional groups present in the bacterial biomass binding Ni^2+^ and Cd^2+^.

Functional Group	Wavelength (cm^−1^)		
	*Pseudomonas aeruginosa 18*	*Enterobacter ludwigii 11Uz*	*Enterobacter cloacae Uz_5*
NH_2_ (amino)	535.26 619.39	535.19	534.97 649.76 619.30
S=O (sulfoxide)	1045.38	1067.55	1049.47
COOH (carboxyl)	1403.79	-	1403.78
N-H (amid)	1567.61	1532.50 1624.16	1558.01
CH (aromatic)	2963.73	-	2963.08
C=O (ketonic)	3278.50	2924.22	3266.92
C-OH (alcoholic)	3278	3276.22	3266.92
P-O (phosphoryl)	-	1235.63	-
C-N (aromatic)	-	1398.99	-
C-H (alkenes)	-	-	925.36

**Table 2:** Capacity of the three bacterial strains to transform Ni^2+^ and Cd^2+^ cations into non-toxic forms.

Strain	Amount of Ni^2+^ and Cd^2+^ Cations Added to the Growth Medium	Indicator Used	Cysteine Formation
*Pseudomonas aeruginosa 18*	Ni^2+^ 2 mM	Na_2_HPO_4_	−
		Na_2_CO_3_	−
		NaOH	−
	Cd^2+^ 3 mM	Na_2_HPO_4_	+
		Na_2_CO_3_	+
		NaOH	+
*Enterobacter ludwigii 11Uz*	Ni^2+^ 3 mM	Na_2_HPO_4_	−
		Na_2_CO_3_	−
		NaOH	−
	Cd^2+^ 1 mM	Na_2_HPO_4_	−
		Na_2_CO_3_	−
		NaOH	−
*Enterobacter* *Cloacae Uz_5*	Ni^2+^ 2 mM	Na_2_HPO_4_	−
		Na_2_CO_3_	−
		NaOH	−
	Cd^2+^ 1 mM	Na_2_HPO_4_	+
		Na_2_CO_3_	+
		NaOH	+
Control	Ni^2+^ 2 mM	Na_2_HPO_4_	+
		Na_2_CO_3_	+
		NaOH	+
	Cd^2+^ 3 mM	Na_2_HPO_4_	+
		Na_2_CO_3_	+
		NaOH	+

* The + sign indicates the formation of the precipitate, while the − sign indicates no precipitate.

## Data Availability

The original contributions presented in the study are included in the article, further inquiries can be directed to the corresponding author.
